# Adapting and implementing very brief advice+ (VBA+) in primary care to promote adolescent smoking cessation in Kyrgyzstan: A mixed-methods FRESHAIR4Life study

**DOI:** 10.18332/tpc/230938

**Published:** 2026-10-23

**Authors:** Julia M. van Koeveringe, Gulzada Mirzalieva, Aijan Taalaibekova, Maamed Mademilov, Azat Bolotbek Uulu, Darush Attar-Zadeh, Luis Carvalho, Rianne MJJ van der Kleij, Nadia Rahim, Purva Abhyankar, Faraz Siddiqui, Talant Sooronbaev, Onno CP van Schayck, Floor A. van den Brand

**Affiliations:** 1 Department of Family Medicine, Faculty of Health, Medicine and Life Sciences, Maastricht University, Maastricht, The Netherlands; 2 Pulmonology Department, National Centre of Cardiology and Internal Medicine, Bishkek, Kyrgyzstan; 3 International Primary Care Respiratory Group (IPCRG), London, United Kingdom; 4 Department of Public Health and Primary Care, Leiden University Medical Center, Leiden, The Netherlands; 5 Division of Psychology, Faculty of Natural Sciences, University of Stirling, Stirling, Scotland; 6 Usher Institute, University of Edinburgh, Edinburgh, Scotland; 7 Department of Health Sciences, University of York, York, United Kingdom; 8 Care and Public Health Research Institute (CAPHRI), Maastricht, The Netherlands

**Keywords:** primary healthcare, implementation science, tobacco cessation, very brief advice plus, adolescent smoking cessation

## Abstract

**INTRODUCTION:**

Tobacco-related morbidity and mortality are increasingly shifting toward low- and middle-income countries (LMICs), with most smokers beginning during adolescence. In Kyrgyzstan, adolescent-specific cessation services are not yet systematically integrated into primary care. This study aimed to culturally and contextually adapt Very Brief Advice Plus (VBA+) to the local context, implement the intervention, and evaluate healthcare professionals’ implementation behaviors and determinants following training.

**METHODS:**

This mixed-methods implementation study was conducted in six Family Medicine Centers in Kyrgyzstan as part of FRESHAIR4Life (2023-2026). VBA+ was adapted for adolescents (aged 14–21 years) through iterative stakeholder reviews to ensure contextual relevance. In total, 56 healthcare providers (HCPs) participated in the study and completed the Determinants of Implementation Behavior Questionnaire (DIBQ) to assess changes in implementation determinants following training on a five-point Likert scale. Qualitative data were collected through semi-structured interviews with 16 HCPs and analyzed using a framework approach, with deductive coding informed by the Theoretical Domains Framework and inductive coding to explore HCPs’ experiences in delivering the intervention.

**RESULTS:**

HCPs initially reported high mean scores of knowledge (3.97) and perceived VBA+ as highly compatible with their professional roles (role and identity; 3.91). At follow-up, construct scores for attitude (mean change= -0.41; p=0.007), reinforcement (-0.57; p=0.019), and memory (-0.52; p=0.023) had decreased significantly, while priority remained the lowest rated determinant (mean=2.61). Qualitative data indicated that training facilitated a shift from authoritarian to empathetic, youth-centered communication. Significant barriers included the presence of parents during consultations and gaps in knowledge regarding emerging products like naswar and e-cigarettes. The use of WhatsApp ‘nudges’ and peer support facilitated sustained implementation.

**CONCLUSIONS:**

This study demonstrates that VBA+ can be feasibly adapted and delivered by trained HCPs in primary care in Kyrgyzstan. Further studies are needed to evaluate its effectiveness and sustainability at a larger scale before broader scale-up can be recommended.

## INTRODUCTION

Tobacco use remains one of the world’s major preventable causes of morbidity and mortality, accounting for over 7 million deaths annually, with an increasing share of tobacco-related harm shifting towards low- and middle-income countries (LMICs)^1,2^. The majority of cigarette smokers begin smoking in adolescence, with early exposure associated with lifelong addiction, reduced life expectancy, and significant physical harm including impaired lung growth and an increased risk of developing chronic respiratory conditions^3,4^. Preventing and addressing tobacco use during adolescence is therefore critical to reducing the future global burden of non-communicable diseases.

Professional cessation support and pharmacological treatment substantially increase the likelihood of successful quitting, whereas most unaided quit attempts fail^5^. Evidence-based non-pharmacological strategies provide individuals with the necessary coping skills and understanding of nicotine addiction to maintain abstinence^6^. Therefore, the World Health Organization identifies primary healthcare as a key setting for tobacco cessation, as routine consultations provide important opportunities to identify tobacco use and deliver brief interventions^7^.

Very Brief Advice (VBA) is an opportunistic, 30-second evidence-based intervention, performed in primary care settings, to refer people who smoke to evidence-based support. It involves three steps: Ask about smoking status, Advise on the best way to quit, and Act by providing referral support to local Stop Smoking Services^8^. As such, services are not widely available, the updated VBA+ framework (Very Brief Advice Plus) acknowledges that patients interested in stopping smoking should be referred to the best locally available support, such as a trained healthcare professional instead of external referral^9^. While VBA is designed for opportunistic delivery in routine care, the current evidence base derives predominantly from adult populations in high-income countries^8,10-12^. Its applicability to adolescents and primary care settings in LMICs remains largely unexplored, representing a significant implementation gap as interventions crafted in high-income countries often prove unsuitable for LMIC contexts without significant modification^13^. Furthermore, the socio-cultural context of a region shapes youth smoking behavior and responses to anti-smoking initiatives, with cultural norms and social expectations influencing intervention outcomes^14^.

Contextually adapted approaches and training are required to integrate adolescent tobacco cessation support systematically into routine primary care, especially as healthcare practitioners (HCPs) frequently report low self-efficacy and significant gaps in knowledge regarding adolescent-specific strategies and novel nicotine devices^15-17^. This need is particularly relevant in Kyrgyzstan, where tobacco control policies have advanced more rapidly than the implementation of cessation support within routine care^18^.

Although Kyrgyzstan ratified the WHO Framework Convention on Tobacco Control in 2006, tobacco use remains a significant contributor to morbidity and mortality, accounting for an estimated 12.9% of all deaths and a smoking prevalence among children aged 10–14 years estimated at 4.4% in 2023^19^. While cessation aids, including nicotine replacement and a national quitline, are available and partially covered by public healthcare, and tobacco control measures such as advertising restrictions, health warnings, and age-of-sale legislation exist, cessation services for adolescents in Kyrgyzstan, as recommended by the WHO, are not yet systematically implemented in primary care^20^.

Family Medicine Centers (FMCs), which serve as the main entry point to healthcare^21^, provide a strategic platform for adolescent tobacco prevention and cessation^22^. As part of the FRESHAIR4Life project, VBA+ was culturally and contextually adapted for adolescents in Kyrgyzstan. This study aims to: 1) describe the adaptation process; and 2) examine healthcare professionals’ implementation behaviors and determinants following VBA+ training.

## METHODS

### Study design

This mixed-methods implementation study is part of FRESHAIR4Life, a four-year (2023–2026) implementation science research project targeting tobacco and air pollution exposure in mid- to late adolescents in disadvantaged populations^23^. In this study, we culturally and contextually adapted VBA+ for adolescent (aged 14–21 years) tobacco cessation in Kyrgyz primary care and evaluated the early implementation of the intervention, including HCPs’ experiences and determinants of delivery following training.

### Setting

The study was conducted in six FMCs in Kyrgyzstan: three in Bishkek and three in the Chui region. The three FMCs in the capital Bishkek each served more than 100000 residents, including many adolescents. Three additional FMCs were recruited from the rural Chui region surrounding Bishkek. Of these, two were larger centers serving approximately 100000 residents each (Chui Sokuluk and Voenno-Antonovka), and one was a smaller center serving around 27000 residents (Kara-Balta).

### Participants

Recruitment of HCPs was conducted through FMCs. The Ministry of Health issued a mandate to FMC directors to facilitate participation in the study. FMC directors identified eligible HCPs providing adolescent care and invited them to participate in VBA+ training and evaluation. Participation was voluntary, and written informed consent was obtained prior to data collection.

### Adaptation of VBA+ intervention

Based on feedback from motivated HCPs and adolescents, intervention materials and training content were adapted to improve relevance for adolescents and the Kyrgyz primary care context. Materials were translated into Russian and Kyrgyz and refined through iterative stakeholder review with HCPs, adolescents, international experts, and national Master Trainers (three pulmonologists from the team: GM, AT, AB). Following pilot testing and stakeholder consultations, the training was redesigned to include interactive learning methods (e.g. discussions, quizzes, and role-play) and was further refined through three pilot deliveries by Master Trainers.

The initial curriculum, based on established VBA materials from the United Kingdom National Center for Smoking Cessation and Training (UK NCSCT)^9^, the Trimbos Institute^24^, and the International Primary Care Respiratory Group (IPCRG)^25^, was refined to ensure contextual and age-specific relevance. Input from champions highlighted key areas that required additional attention, including: 1) practical explanations of both the short- and long-term effects of nicotine and tobacco use; 2) the harms and addictive potential of electronic cigarettes; and 3) communication strategies tailored to adolescents.

Materials included an HCP guide, youth-oriented brochures and posters, and visual aids to prompt and support delivery, all translated into Russian and Kyrgyz and iteratively refined through multiple rounds of review and adaptation involving both the scientific team and adolescents.

### Training of Master Trainers and HCPs

Training was delivered in two stages. In November 2024, the Master Trainers completed a ‘Teach the Teacher’ program delivered by an international expert (DA), consisting of three online sessions on working with adolescents, VBA+ and co-design approaches. Second, the three Master Trainers delivered VBA+ training to HCPs over two sessions. A WhatsApp group was created to continue peer support and learning. In the months after training, this platform was used to send out nudges to remind HCPs to conduct VBA+. A research assistant took structured field notes, and international researchers (FvdB, OvS) were present to observe and provide feedback to the Master Trainers.

### Questionnaires

The Theoretical Domains Framework (TDF) was used as the overarching framework to guide both quantitative and qualitative data collection. Quantitatively, implementation determinants were assessed using the Determinants of Implementation Behavior Questionnaire (DIBQ), a 93-item self-report tool measuring 18 domains of the TDF related to implementation behavior^26^. This DIBQ was adapted for VBA delivery in Kyrgyz primary care and supplemented with questions specifically aimed at using a youth-centered approach (see Supplementary file). Items were scored on a five-point Likert scale; higher scores indicate stronger agreement or more favorable perceptions of the determinant.

### Interviews

Between 6 August and 18 December 2025, 16 potential participants were invited to participate in an individual interview. The semi-structured interview guide explored healthcare professionals’ experiences of delivering VBA+ across selected domains of the TDF, aligned with the DIBQ. These included: 1) beliefs about adolescent tobacco and nicotine use and perceived relevance and acceptability of VBA+ to professional role and values; and 2) experiences with intervention delivery, including training, use of implementation materials, and confidence in delivering brief advice and follow-up consultations.

### Data collection

After the first training (T1), all participating HCPs completed a demographic questionnaire and the DIBQ. Demographic characteristics included place of work (urban, rural), gender (female, male), age (21–34, 35–54, 55–75 years), professional role (general practitioner, nurse, other), years of working experience (<1, 1–3, 4–6, >6), years of experience working with youth (none, <1, 1–3, 4–6, >6), previous tobacco/e-cigarette use (yes, no, and past 30-day use), prior experience delivering VBA+ and experience providing smoking cessation counselling (none, minimum, some, significant, great).

The implementation period started after the first training and ran from March to September 2025. After the second training session (T2) in March, HCPs completed follow-up DIBQ assessments and a training evaluation.

Qualitative data were collected through individual face-to-face in-depth interviews in the local Kyrgyz language between 6 August and 18 December 2025. HCPs recorded each VBA+ delivery using standardized reporting forms, which were used to identify differences in intervention uptake and continued delivery over the 3-month follow-up period for qualitative sampling. Members of the research team recruited HCPs using purposive maximum variation sampling to ensure representation across professional cadres (general practitioners, nurses, and other staff) and varying levels of VBA+ implementation. Interviews were conducted in Kyrgyz and Russian at FMCs and at the participants’ workplaces. Team members (GM, AT, AB, MM) conducted the interviews and were trained by FS. Considering the local context and dialect, all interviews were accurately transcribed verbatim and translated into English.

### Data analysis

Quantitative DIBQ data were analyzed to assess changes in implementation determinants. Domain-level descriptive statistics were calculated at T1 and T2, and paired-samples t-tests examined changes in scores between time points. Because some variables deviated from normality and the sample size was modest, bootstrapping procedures with 2000 bias-corrected and accelerated (BCa) samples were applied to obtain robust confidence intervals. All quantitative analyses were performed in IBM SPSS Statistics version 29, using a two-tailed alpha level of 0.05 to determine statistical significance.

A public health scientist (JvK), trained in qualitative methods and qualitative analysis, conducted the primary analysis. JvK was not involved in DIBQ data analysis prior to coding to minimize interpretive bias. Qualitative data from semi-structured interviews were analyzed thematically in ATLAS.ti.

Qualitative data were analyzed using a framework approach. An initial coding framework was developed deductively from selected TDF domains and expanded inductively to capture emergent barriers and facilitators. Codes were iteratively refined and organized into themes representing determinants of VBA+ implementation. Interpretations were discussed between two researchers (JvK, FvB), focusing on consistency and relevance in relation to implementation processes.

Given the dual role of interviewers as VBA+ trainers, participants were aware of the researchers’ involvement in the intervention training, which was considered during interpretation of findings. Reflexivity was addressed through the maintenance of reflexive memos throughout data collection and analysis, and through iterative discussions within the research team. Representative quotations were included to illustrate the interpretation of the themes.

### Ethics

The study protocol was approved by the Ethics Committee of the National Center of Cardiology and Internal Medicine, Kyrgyzstan, and by the University of York Research Governance Committee (Protocol No. 4; 12.05.2023). All HCPs provided written informed consent prior to participation. All data were pseudonymized prior to analysis, and confidentiality was maintained throughout. Participants did not receive financial incentives, but travel expenses were reimbursed and refreshments provided.

## RESULTS

### Participant characteristics

A total of 56 HCPs participated in the first round of VBA+ training in February 2025, including 33 in Bishkek and 23 in Chui. The second round, held in March 2025, included 38 participants (21 in Bishkek and 17 in Chui). Participants consisted of 20 general practitioners, 28 nurses, and 8 other professionals, including health promotion specialists and school nurses, with a median of 4–6 years of clinical experience in adolescent care (range: 14–21 years). Three participants were unable to attend the second training round due to clinical responsibilities or scheduling conflicts with other training activities. Additional demographic and professional characteristics are summarized in Table 1.

**Table 1:** Baseline characteristics of healthcare professionals participating in the Very Brief Advice+ training program, Kyrgyzstan, 2025 (N=56)

*Characteristics*	*n*	*%*
**Data collected**		
Baseline only	15	26.8
Follow-up only	18	32.1
Baseline and follow-up	23	41.1
**Place of work**		
Urban	33	58.9
Rural	23	41.1
**Gender**		
Male	2	3.6
Female	54	96.4
**Age** (years)		
21–34	21	37.5
35–54	17	30.4
55–75	18	32.1
**Job title**		
General practitioner	20	35.7
Nurse	28	50
Other	8	14.3
**Work experience** (years)		
<1	5	8.9
1–3	8	14.3
4–6	7	12.5
≥7	36	64.3
**Years of experience working with youth aged 14–21 years**		
None	8	14.3
<1	8	14.3
1–3	6	10.7
4–6	8	14.3
≥7	26	46.4
**Have you tried using tobacco products at least once or twice?**		
No	49	87.5
Yes	7	12.5
**During the last 30 days, on how many days did you smoke cigarettes?**		
0	55	98.2
1–2	1	1.8
**Have you ever tried using e-cigarettes or vapes, even once or twice?**		
No	54	96.4
Yes	2	3.6
**Over the past 30 days, on how many days have you used e-cigarettes or vapes?**		
0	55	98.2
1–2	1	1.8
**Experience delivering VBA+**		
None	27	48.2
Minimum	22	39.3
Some	3	5.4
Significant	3	5.4
Great	1	1.8
**Experience providing smoking cessation counseling**		
None	8	32.1
Minimum	28	50.0
Some	5	8.9
Significant	4	7.1

### Quantitative evaluation of HCP training outcomes

The results of the HCP training, as measured by the DIBQ, are detailed in Table 2.

**Table 2:** Changes in implementation determinants among healthcare professionals following Very Brief Advice Plus (VBA+) training in six Family Medicine Centers in Bishkek and the Chui region, Kyrgyzstan, February–March 2025 (paired observations, N=23)

	*After Training 1 Mean (SD)* *(N=38)*	*After Training 2 Mean (SD)* *(N=35)*	Mean difference (95% BCa CI)* *(N=23)*	*p*
**Knowledge**	3.97 (0.09)	3.78 (0.13)	-0.185 (-0.500–0.084)	0.264
Understanding VBA+ components and youth health risks				
**Youth-centered approach**	3.89 (0.10)	3.67 (0.15)	-0.217 (-0.446 – -0.003)	0.103
Using relatable language and providing tailored advice				
**Attitude**	3.90 (0.13)	3.49 (0.13)	-0.406 (-0.652 – -0.188)	**0.007**
Perceiving VBA+ delivery as easy, useful and worthwhile				
**Self-efficacy VBA**	3.69 (0.10)	3.70 (0.14)	0.014 (-0.203–0.232)	0.902
Confidence in initiating conversations and delivering steps				
**Self-efficacy follow-up**	3.75 (0.12)	3.62 (0.14)	-0.130 (-0.450–0.159)	0.437
Confidence in conducting consultations				
**Innovative characteristics**	3.97 (0.08)	3.70 (0.14)	-0.275 (-0.565 – -0.029)	0.080
Perceived simplicity, accessibility and compatibility of VBA+				
**Outcome expectancies**	3.67 (0.12)	3.65 (0.15)	-0.022 (-0.413–0.304)	0.915
Belief in clinical impact and patient appreciation				
**Patient characteristics**	3.28 (0.11)	3.52 (0.15)	0.239 (-0.104–0.543)	0.221
Perceiving youth as positive and motivated				
**Action planning**	3.65 (0.13)	3.57 (0.15)	-0.087 (-0.326–0.174)	0.576
Concrete plans for delivery and overcoming barriers				
**Organization resources and support**	3.51 (0.13)	3.40 (0.15)	-0.109 (-0.500–0.272)	0.586
Availability of physical materials and institutional backing				
**Implementation needs**	3.07 (0.17)	3.25 (0.20)	0.174 (-0.725–0.377)	0.531
Requirements for additional training/information				
**Maintain positive**	3.87 (0.46)	3.61 (0.78)	-0.261 (-0.609–0.043)	0.177
Staying inspired despite barriers				
**Role and identity**	3.91 (0.67)	3.57 (0.79)	-0.348 (-0.696 – -0.011)	0.070
Sense of professional duty to deliver VBA+				
**Intention**	3.83 (0.49)	3.70 (0.82)	-0.130 (-0.514–0.217)	0.517
Stated commitment to deliver VBA+ soon after training				
**Priority**	2.61 (0.89)	2.74 (0.75)	0.130 (-0.217–0.522)	0.531
Importance compared to other tasks				
**Memory**	3.43 (0.84)	2.87 (1.01)	-0.565 (-0.957 – -0.174)	**0.023**
Frequency of forgetting to initiate VBA+				
**Reinforcement**	3.96 (0.56)	3.43 (0.79)	-0.522 (-0.826 – -0.217)	**0.019**
External appreciation received for executing VBA+				

All determinants were measured on a 1–5 Likert scale, with higher scores indicating stronger agreement or more favorable perceptions of the determinant. Mean differences reflect change scores computed as T2 - T1, such that positive values indicate an increase from baseline to follow-up.

* BCa: bias-corrected and accelerated. CI: confidence interval. Paired-samples t-tests with 2000 bootstrap samples were used to estimate (BCa) 95% CI. P-values are based on bootstrapped two-tailed significance tests. See Supplementary file for more detailed explanation of the constructs.

In general, HCPs reported high mean construct scores across most domains, with baseline means typically exceeding 3.5. At baseline, the highest rated mean scores were knowledge and innovative characteristics (both mean=3.97), indicating that providers initially felt they understood the program well and viewed it as highly compatible with their work. The lowest mean score was observed for priority (mean=2.61), indicating that tobacco cessation was perceived as a relatively low priority compared with other clinical responsibilities. The data showed significant decreases in attitude (mean change= -0.41; 95% BCa CI: -0.65 – -0.19; p=0.007), reinforcement (mean change= -0.52; 95% BCa CI: -0.83 – -0.22; p=0.019), and memory (mean change= -0.57; 95% BCa CI: -0.96 – -0.17; p=0.023) at follow-up.

### Qualitative evaluation: barriers and facilitators related to providing VBA+

Of the 16 participants, one was male, and fifteen were female. Four participants were from rural centers, and twelve from urban settings. Six nurses and ten GPs participated. The interview length ranged from 14 to 42 minutes. The following qualitative themes were mapped directly onto TDF domains to facilitate interpretation of changes in HCPs’ behavior following training.

### Knowledge

HCPs noted that their knowledge of tobacco’s harms was essential for communicating risks effectively. Before training, practitioners often face challenges when patients ask for details regarding the chemical composition of various products such as traditional tobacco, e-cigarettes, or naswar (a type of moist, powdered, smokeless tobacco). One professional explained:

*‘We’re getting more questions from patients, and we can’t answer some of them. For example, what’s tobacco made of? … The same goes for naswar, we can’t always give a definitive answer to what it’s made of.’* (GP3, FMC2, rural)

There was a specific need to debunk myths prevalent among youth, such as the idea that vapes are harmless. Professionals suggested that having more information on specific conditions, like *‘popcorn lung disease’* (GP8, FMC4, urban), would help them inform the younger generation more effectively.

### Skills

HCPs felt that VBA+ training enabled a transition from an authoritarian approach to a more sensitive and empathetic one. HCPs acknowledged that authoritarian demands to quit frequently resulted in patient disengagement or secrecy:

*‘After training, we became more gentle and found a way to approach adolescents. Before, we were harsh and demanded they quit smoking, but now we have a different approach.’* (GP3, FMC2, rural)

Practicing through roleplays during training helped address initial discomfort with discussing smoking, a topic that many HCPs were not used to raising during routine patient encounters. The exercises also provided professionals with specific phrases to use in common situations. Participants mentioned they ‘mastered’ these communications over time, becoming more comfortable in asking out information from the quiet youth who might otherwise avoid the subject. Providers emphasized the importance of finding a ‘common language’ to discuss sensitive topics more gently:

*‘I tried to find a common language, to explain these points about the dangers of tobacco more gently and in more detail. Little by little we managed to find a common language.’* (GP1, FMC4, urban)

### Self-efficacy

Participants mentioned that they become more confident over time, resulting from a combination of training and real-world practice:

*‘[The training made me] more confident than usual. Because we also learned in training how to approach adolescents, how to talk to them, how to begin interviewing them. Because asking them directly is uncomfortable for them. If they use tobacco, they can say, “No, I don’t smoke”. We also covered these issues in training sessions.’* (GP9, FMC4, urban)

### Outcome expectancies

Due to the training, several doctors came to recognize that clinical success is not static or solely defined by quit rates. While complete cessation remained the ultimate goal, they also valued initiating dialogue or influencing a young person’s views as a ‘small victory’, which made subsequent consultations feel more manageable:

*‘At first, I was afraid … Then I somehow realized that my task was this: yes, if he quits smoking, that’s great … But if I can at least change this young person’s opinion, that’s already a small victory. And after that, it became a little easier.’* (GP11, FMC4, urban)

### Social/professional role and identity

The motivation to conduct VBA was deeply tied to the participants’ sense of professional duty and their role as the primary point of contact in the healthcare system. HCPs mentioned they were unfamiliar with smoking cessation programs for adolescents before the training:

*‘Usually nobody dealt with this before. Of course, we talked about smoking in general, but adolescents were always overlooked … this training teaches not only how to notice it, but also how to help them get rid of this dependence on smoking.’* (GP6, FMC1, urban)

Practitioners view they bear a specific responsibility to protect the younger generation from the long-term harms of both traditional cigarettes and modern electronic nicotine products:

*‘I suppose, VBA was conceived in such a way that family doctors conduct the initial consultation. We are the primary healthcare structure … Therefore, I think we are responsible for protecting young people from harmful substances such as tobacco smoking or electronic cigarettes.’* (GP11, FMC4, urban)

Another professional noted:

*‘Well, since we work with this problem [smoking addiction], we are obliged. It is kind of our duty, our usual practice, you can’t escape from this.’* (GP4, FMC3, rural)

### Social influences

While some HCPs integrated VBA+ immediately, others were more hesitant and relied heavily on peer support and collective learning to overcome their initial fears:

*‘We were afraid to start … We started asking each other, calling each other, asking those who had been trained how to start, how they were doing it.’* (GP3, FMC2, rural)

### Memory, intention, and priority

A common barrier was the overwhelming daily workload, which caused the new intervention to be deprioritized. Without regular repetition, some practitioners forgot to initiate VBA when they encountered a young patient:

*‘Over time, my motivation decreased … Probably because we didn’t do it every day … Sometimes we even forget about it. We remember when we see an adolescent, but otherwise it’s not on our minds every day because we don’t use it.’* (Nurse6, FMC1, urban)

To counter this fading, external reminders, including nudges through WhatsApp from Master Trainers and the developed desktop bars, were found to be crucial. One medical worker shared how group communication helped maintain consistency:

*‘Maybe it will sound funny, but at the beginning we often forgot about consultations because of the main work. And our coordinators [Master Trainers] constantly wrote to us in the chat: “Don’t forget, don’t forget”. Thanks to their responsibility it became a habit’*. (GP1, FMC4, urban)

### Environmental context and resources

The presence of parents emerged as one of the greatest social barriers to the effective delivery of VBA+, as parents’ involvement was perceived to limit adolescents’ openness and willingness to disclose smoking-related behavior. To mitigate this, HCPs developed ‘privacy-seeking’ strategies and learned the skills to create the opportunity for conducting VBA+ while practicing, such as sending parents to the cashier or pharmacy, to have a few minutes of confidential time with the adolescent:

*‘Well, we usually send them to the cashier, to pay, or somewhere else to wait in line. We take advantage of those moments’*. (GP7, FMC1, urban)

Additionally, HCPs recommended expanding the training to settings where adolescents come more often, so more adolescents can be reached:

*‘We need to expand our reach to family medicine centers, family doctors’ offices, and school clinics, as we have a nurse in every school, and they can be involved in teaching. Plus, universities, as a large number of students also smoke, so we can conduct these trainings and seminars there.’* (GP1, FMC4, urban)

### Attitude and reinforcement

HCPs viewed the training as useful as it provided them with a clear and structured algorithm for conducting smoking cessation counseling, which simplified clinical practice. VBA training reinforced perceived capability and confidence by translating the intervention into an easy-to-apply routine:

*‘I consider that this is a necessary program in conditions of strong societal dependence on tobacco products. It simplifies the process of counseling patients with tobacco dependence: in the head a clear algorithm is built of how to properly conduct a conversation with such a person.’* (GP8, FMC4, urban)

Despite high levels of interest, motivation was subject to fluctuations, with enthusiasm often fading after a few months:

*‘At the beginning, yes, it influenced positively … Later, it gradually faded … After about three to four months. Since I was not deeply engaged in it, enthusiasm gradually faded.’* (GP6, FMC1, urban)

### Behavioral regulation

Ultimately, repetition and regularity were key to turning VBA into a habit. Professionals found that once they tried the method a few times, they realized it was not as ‘scary’ as they had feared and were able to develop their own personal techniques for asking the right questions. Besides practicing during consultations, HCPs indicated that they would value more training and repetition, as a refresher and to follow the latest trends:

*‘I’ll probably continue. We work in healthcare, and we need to protect our children for the future, so we need the VBA program. If there are more trainings and seminars, we’re ready to continue this consulting.’* (GP10, FMC6, urban)

## DISCUSSION

This mixed-methods study presents the adaptation and early implementation of the Very Brief Advice Plus (VBA+) approach for adolescents in a primary care context in Kyrgyzstan. Overall, HCPs viewed adolescent tobacco cessation counseling positively and considered VBA+ a relevant and practical approach for routine care. The DIBQ findings indicated that HCPs reported favorable scores on most determinants related to the delivery of VBA+. However, after an initial trial period of four weeks of practicing VBA+ in their setting, HCPs scored lower on perceiving VBA delivery as easy, useful, or worthwhile and were more likely to report forgetting to initiate VBA+ and needing external appreciation for delivering it. These changes may indicate that the training and early implementation phase increased HCPs’ awareness of practical implementation barriers that had not been fully anticipated beforehand, including the need for prompts, reinforcement, and integration into routine workflows. As such, lower post-training scores may represent increased implementation insight, rather than reduced acceptability of VBA+.

During the interviews, HCPs reported limited exposure to adolescent patients within their centers, which reduced the opportunity for repeated delivery and skill practice, thereby hindering the VBA+ intervention from becoming habitual. At the same time, HCPs consistently reported that repeated practice, both during roleplay sessions during training and real-world consultations, enhanced their confidence and perceived competence in delivering the intervention, thereby highlighting the importance of ongoing training and reinforcement. In addition, HCPs demonstrated foundational knowledge regarding the harms of tobacco use, while also identifying specific uncertainties related to emerging nicotine products, including electronic cigarettes and naswar. These knowledge gaps occasionally reduced confidence when responding to adolescents’ questions, suggesting a need for continued training focused on youth-specific and evolving tobacco products.

The findings align with existing literature highlighting that formal training and continued support are necessary for intervention delivery to become integrated and habitual^8^. Similarly, van Schayck et al.^27^ describe how VBA shifted GPs from a ‘preaching’ role to a more patient-centered, supportive approach, lowering perceived confrontation and easing initiation of cessation conversations. However, sustaining such behavioral changes remains challenging over time, and the evidence on the long-term impact of smoking cessation interventions is still limited^28^. Research in Sub-Saharan Africa confirms that while training improves immediate self-efficacy, systemic support, such as upgrading clinical information systems for better documentation, is essential for the proactive, habitual delivery of cessation support^29^. Our study’s reliance on external WhatsApp ‘nudges’ to maintain implementation fidelity aligns with these findings, demonstrating that structural reminders are vital for habit formation in busy primary care environments. Furthermore, literature highlights the need for regular training as a solution to keep professional assumptions and knowledge on emerging challenges up to date^30^.

The WHO recommends that brief advice be consistently provided by healthcare providers as a routine practice to all tobacco users accessing any healthcare settings^7^. However, evidence shows that in practice, healthcare professionals may forget to deliver cessation advice when consultations are focused on unrelated or acute complaints, reflecting competing demands and priorities in primary care workflows^27^. This aligns with our findings in the Kyrgyz setting, where limited adolescent consultation frequency and competing clinical priorities likely contributed to reduced prioritization and routine delivery of VBA+. Introducing modest incentives or formal recognition for active implementers may further reinforce engagement, particularly in busy primary care environments^31^.

This study also highlights the importance of collaborative working between the research team, HCP champions, local government stakeholders and the target population in supporting effective implementation. The use of co-design enabled the training and intervention materials to be refined to meet HCP needs, improve communication with adolescents and support intervention delivery. These findings align with evidence that grounding interventions in real-world contexts enhances implementation success^32^.

### Strengths and limitations

While previous research on VBA and similar brief advice models has primarily focused on adult populations in high-income countries^33^, this study extends the approach to adolescents aged 14–21 years. It illustrates that primary care-based brief interventions can be effectively adapted for adolescents when content and delivery are aligned with their development stage and local sociocultural context.

However, the findings should be interpreted in light of limitations such as the short follow-up period, small sample size, reliance on self-reported DIBQ data, and inclusion of only six FMCs in the Chui region and Bishkek, which may limit generalizability. However, the paired design accounts for stable differences between participants; time-varying contextual factors could not be accounted for. In the absence of a comparison group, the observed changes therefore cannot be causally attributed to the training or implementation of VBA+. Future studies should evaluate the effectiveness and implementation of VBA+ over a longer period, while also examining adolescents’ long-term behavior change and perceptions of the counseling process.

## CONCLUSIONS

Conducted in rural and urban settings in the Kyrgyz Republic, this intervention demonstrates that, with appropriate contextual adaptation, VBA+ can be feasibly delivered by trained healthcare professionals. Although most tobacco use begins in adolescence, cessation support for young people in low-resource settings remains limited. Our findings highlight the potential of contextually adapted, evidence-based training for healthcare providers in primary care to support delivery of tobacco cessation counseling. Future studies should evaluate the implementation of VBA+ in adolescent primary care settings at a larger scale and over longer follow-up periods to identify context-specific strategies for its successful integration into routine clinical practice.

## Supplementary Material



## Data Availability

The data supporting this research are available from the authors on reasonable request.
