# Supplementary material for: Adapting and implementing very brief advice+ (VBA+) in primary care to promote adolescent smoking cessation in Kyrgyzstan: A mixed-methods FRESHAIR4Life study
Source: Tob Prev Cessat. 2026 Oct 23;12:10.18332/tpc/230938. doi: 10.18332/tpc/230938 (PMC13693424; doi:10.18332/tpc/230938)
Supplement: Supplementary file 1 [file TPC-12-67-s1.pdf]

TOBACCO PREVENTION AND CESSATION

Supplementary file

© 2026 van Koeveringe J.M. et al.

DOI:
10.18332/tpc/230938

The content has been provided by the author(s) and has not been reviewed, verified, or endorsed by European Publishing. It may not have undergone peer review. The views, opinions, and recommendations expressed are solely those of the author(s) and do not necessarily reflect the position of European Publishing. European Publishing accepts no responsibility or liability for any consequences arising from the use of, or reliance on, this content.

Appendix I. DIBQ Constructs and Questionnaire Items

This document provides an overview of the constructs assessed in the adapted Determinants of Implementation Behaviour Questionnaire (DIBQ) and the questionnaire items belonging to each construct.

Knowledge

I understand the basic components of the VBA+ smoking cessation program.

I am aware of the specific risks of smoking for young patients (e.g. long-term health effects, addiction).

I can explain the immediate benefits of quitting smoking in a way that young patients can understand

I have sufficient knowledge to conduct VBA+ classes according to the manual.

Skills

I have sufficient skills to perform VBA+ as directed.

I can effectively engage young people in conversations about smoking without judgment.

I assess young people's progress in quitting smoking and provide additional guidance when necessary.

Youth-centred approach

I use language and examples that are relevant and understandable to young patients

I take into account the special difficulties that young people face when trying to quit smoking.

I encourage young people to express their thoughts, feelings and concerns about smoking and quitting.

I give individual advice that matches the interests, habits and motives of young people.

Attitude

For me, delivering VBA+ following the guidelines is….(easy)

For me, delivering VBA+ following the guidelines is….(useful)

For me, delivering VBA+ following the guidelines is….(worthwhile)

Self-efficacy (VBA delivery)

I feel confident when initiating a conversation about smoking cessation with young patients.

I confidently ask young patients about their smoking habits at every appointment.

I am confident that I can provide VBA+ following the instructions.

I am confident that I will be able to carry out VBA+ according to the recommendations, even if I encounter obstacles.

I feel confident when I ask a young mans parents to wait in the waiting room during a consultation.

I feel confident discussing the topic of smoking in the presence of a young person if his parent is present during the consultation.

Self-efficacy (follow-up consultation)

I feel confident recommending resources to young people (websites, apps).

I feel confident when I conduct follow-up consultations with young people to discuss how to quit smoking.

I confidently offer young people behavior change methods to help them quit smoking.

<u>Innovative characteristics</u>

VBA+ can be adapted to the needs of young people.

The use of VBA+ for young people is compatible with everyday practice.

VBA+ is simple and accessible.

<u>Outcome expectancies</u>

If I use VBA+ as recommended, young people will appreciate it.

I If I carry out VBA+ as recommended, it will help young people quit smoking.

<u>Patient characteristics</u>

Young patients generally respond positively to VBA+.

Young patients are motivated to receive advice on smoking cessation.

<u>Action planning</u>

I have a clear plan for how I will perform VBA+ for young patients according to the recommendations.

I have a clear plan for how I will deliver VBA+ to young patients, following guidelines when I encounter barriers.

<u>Organisational resources and support</u>

My organisation has all the necessary resources (e.g. equipment, materials, space) to provide VBA+ to young patients.

My organisation, has formal policies regarding the use of VBA+ (e.g. strategies, work programs, etc.)

My organization leadership provides with sufficient support in conducting the VBA+ programme for young people.

I can count on support from the trainers with whom I implement VBA+ when difficulties arise in implementing VBA+ for young people.

<u>Implementation needs</u>

I need additional information to use VBA+ correctly.

I need additional training to properly use VBA+.

I need additional support to use VBA+ correctly.

Single-item constructs

<u>Maintain positive</u>

I maintain a positive and inspiring attitude even when young people resist change.

<u>Role and identity</u>

It is my responsibility as a healthcare provider to deliver VBA+ following the guidelines.

<u>Intention</u>

I will definitely deliver VBA+ to young patients following the guidelines in the next three months.

<u>Priority</u>

How often is working on something else on your agenda a higher priority than delivering VBA+ to young patients?

<u>Memory</u>

Delivering VBA+ to young patients is something I often forget.

<u>Reinforcement</u>

I receive sufficient appreciation for the execution of VBA+.
